# Large-scale monitoring of effects of clothianidin-dressed OSR seeds on pollinating insects in Northern Germany: effects on large earth bumble bees (*Bombus terrestris*)

**DOI:** 10.1007/s10646-016-1730-y

**Published:** 2016-09-27

**Authors:** Guido Sterk, Britta Peters, Zhenglei Gao, Ulrich Zumkier

**Affiliations:** 1 IPM Impact, Gierkensstraat 21, Kuringen, 3511 Belgium; 2 tier3 solutions GmbH, Leverkusen, Germany

**Keywords:** Bumble bees, Seed treatment, Plant protection products, Neonicotinoids

## Abstract

The aim of this study was to investigate the effects of Elado^®^-dressed winter oilseed rape (OSR, 10 g clothianidin & 2 g beta-cyfluthrin/kg seed) on the development, reproduction and behaviour of large earth bumble bees (*Bombus terrestris*) as part of a large-scale monitoring field study in Northern Germany, where OSR is usually cultivated at 25–33 % of the arable land. Both reference and test sites comprised 65 km^2^ in which no other crops attractive to pollinating insects were present. Six study locations were selected per site and 10 bumble bee hives were placed at each location. At each site, three locations were directly adjacent to OSR fields and three locations were situated 400 m distant from the nearest OSR field. The development of colonies was monitored from the beginning of OSR flowering in April until June 2014. Pollen from returning foragers was analysed for its composition. An average of 44 % of OSR pollen was found in pollen loads of bumble bees indicating that OSR was a major resource for the colonies. At the end of OSR flowering, hives were transferred to a nature reserve until the end of the study. Colony development in terms of hive weight and the number of workers showed a typical course with no statistically significant differences between the sites. Reproductive output was comparatively high and not negatively affected by the exposure to treated OSR. In summary, Elado^®^-dressed OSR did not cause any detrimental effects on the development or reproduction of bumble bee colonies.

## Introduction

Pollination is one of the most essential ecosystem services provided by nature not only to wild plant species, but also for a number of arable crops (Klein et al. 2007). However, several studies suggest that there is a decline in populations of pollinating insects (Kearns et al. 1998; Biesmeijer et al. 2006; Potts et al. 2010). While domesticated honey bees are traditionally thought of as the economically most important pollinator in crop monocultures, bumble bees can also be important pollinators especially in temperate climates (Free 1970; Corbet et al. 1991). While commercially bred colonies are available for the use in agriculture (Velthuis and Doorn 2006), their use is often restricted to greenhouses so that field crops rely on naturally occurring bumble bee colonies.

However, declines in populations of bumble bees have been reported worldwide (e.g., Kosior et al. 2007; Williams and Osborne 2009; Colla et al. 2012; Kerr et al. 2015). Multiple stressors may affect bumble bees, e.g., parasites, lack of floral resources and plant protection products (PPPs) (Goulson et al. 2015). In agricultural landscapes, mass-flowering crops such as OSR serve as a valuable nectar and pollen source for bumble bees (Westphal et al. 2006 2009), but, on the other hand, agricultural practices such as the use of PPPs may pose a risk to pollinating insects.

A class of PPP that has been commonly used in OSR are neonicotinoids. Formulations containing neonicotinoids may be used as a seed treatment; their active substances are systemically taken up by the plants and distributed to all tissues (Elbert et al. 2008). The use of seed dressing reduces risks for non-target organisms, as fewer applications at lower rates are used as compared to foliar spray applications. However, concerns have been raised regarding the exposure of flower visiting insects due to the potential presence of the substances in nectar and pollen (Blacquière et al. 2012). Due to these concerns the use of the three neonicotinoids imidacloprid, clothianidin and thiamethoxam was temporarily suspended in the European Union in crops attractive to bees (European Commission 2013).

Various laboratory and semi-laboratory experiments have been performed, where bumble bees were artificially exposed to ‘field-realistic concentrations’ of neonicotinoids (e.g., Whitehorn et al. 2012; Feltham et al. 2014). However, whether concentrations used in these experiments are really representative for the exposure in the field is still under debate (Carreck and Ratnieks 2014). Furthermore, existing laboratory studies for clothianidin (e.g., Franklin et al. 2004; Scott-Dupree et al. 2009; Scholer and Krischik 2014; Moffat et al. 2015) are inconsistent in their implications for bumble bee colonies in the field.

Thus, a key question is how neonicotinoids influence bees in real world agricultural landscapes (Schmuck and Lewis 2016). However, only few monitoring studies at the landscape level have been performed (e.g., Cutler and Scott-Dupree 2014; Thompson et al. 2016). To our knowledge, only two studies of bumble bees exposed to clothianidin treated OSR exist. An attempt has been made to assess the impact of neonicotinoids on bumble bee colonies under field conditions by UK’s Food & Environment Research Agency (Thompson et al. 2013). While the authors stated that there was no clear relationship between the use of neonicotinoids and colony performance a recent re-evaluation (using different statistics) came to the opposite conclusion (Goulson 2015). Rundlöf et al. 2015 reported a negative effect on bumble bee colony development and reproduction in a field study with clothianidin-treated spring OSR in Sweden.

Despite their role in ecology and the high economic value of their pollination services, bumble bees are not part of the testing regime routinely used for the registration of PPPs. Only recently, bumble bees testing and the associated risk assessment have been implemented in a new guidance document (EFSA 2013) which has, however, not yet come into force. In contrast to the well-established tests on honeybees following the usual tiered approach from worst-case laboratory testing to most realistic testing under field conditions, no validated methodology for PPP testing on bumble bees exists. Our aim was to use methods that are not only comprehensive, but also suitable to monitor effects on bumble bees taking into account their unique biology. We believe that a number of factors are important to achieve meaningful results.

Firstly, hives should be standardized and the queens should be from the same hibernation batch. It is a common misunderstanding that commercial bumble bee hives are very homogenous in their composition. Quite often, number and age of workers are not similar between colonies as they are prepared to accommodate the requirements of different crops. Surplus hives are then sold in mixed batches. This can also lead to differences in queen age and quality, both of which have a significant influence on further colony development. A second crucial point is the method chosen to determine the reproductive success. Bumble bees do not overwinter as entire colony, but young queens hibernate individually to found new colonies in the next spring. The onset of the production of new queens (the so-called turning point) marks an important event in the life cycle of a bumble bee colony. However, ecotoxicological experiments should not be terminated when the first colonies reach the turning point. According to our experience, the number of queens produced is even more important than the time of the turning point. For the assessment of reproduction success, not only should the number of new queens be counted, but also the number of queen cells produced should be taken into account. Additional information regarding the use of bumble bees in ecotoxicological studies can be found in Sterk et al. (2002); Mommaerts et al. (2009) and Mommaerts et al. (2010). Furthermore, Cabrera et al. (2016) give valuable recommendations for higher-tier assessments on bumble bees.

The aim of this monitoring study at the landscape level was to test the potential impact of winter OSR grown from clothianidin-treated seeds on bumble bees under the most realistic field conditions (winter OSR planted in the previous year treated with a product that was registered for use in Germany before the current moratorium). Potential effects on survival, development and reproduction of bumble bees were assessed in colonies placed directly adjacent or at a distance (ca. 400 m) from fields of flowering OSR.

## Materials and Methods

### Test species

The test species *Bombus terrestris dalmatinus* has its origin in Turkey and is the commercially most widely used subspecies in Western Europe (Velthuis and Doorn 2006). Colonies for the study were obtained from Koppert Biological Systems (Berkel en Rodenrijs, The Netherlands). Transportation of bumble bee colonies was always carried out in a refrigerated truck.

### Study location and design

**Fig. 1:**
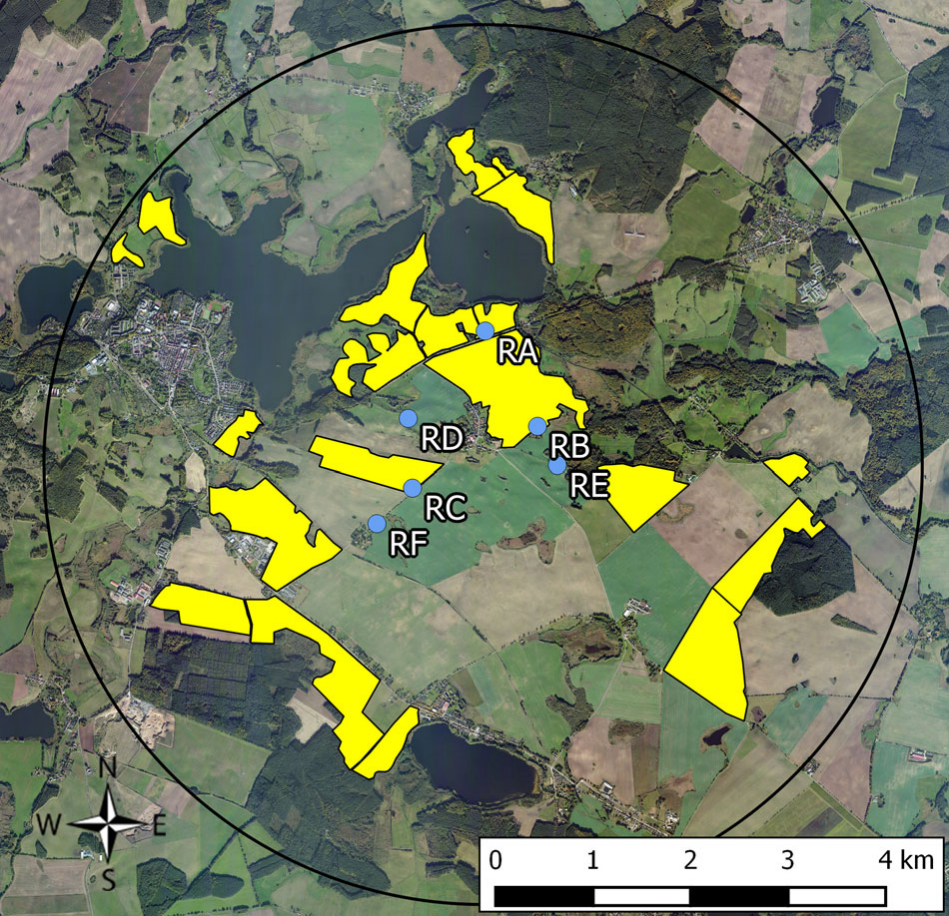
Reference site (fields with untreated seeds) used for the monitoring of effects of flowering OSR grown from clothianidin-dressed seeds. Study locations are marked in *blue*, *yellow polygons* indicate the study fields

**Fig. 2:**
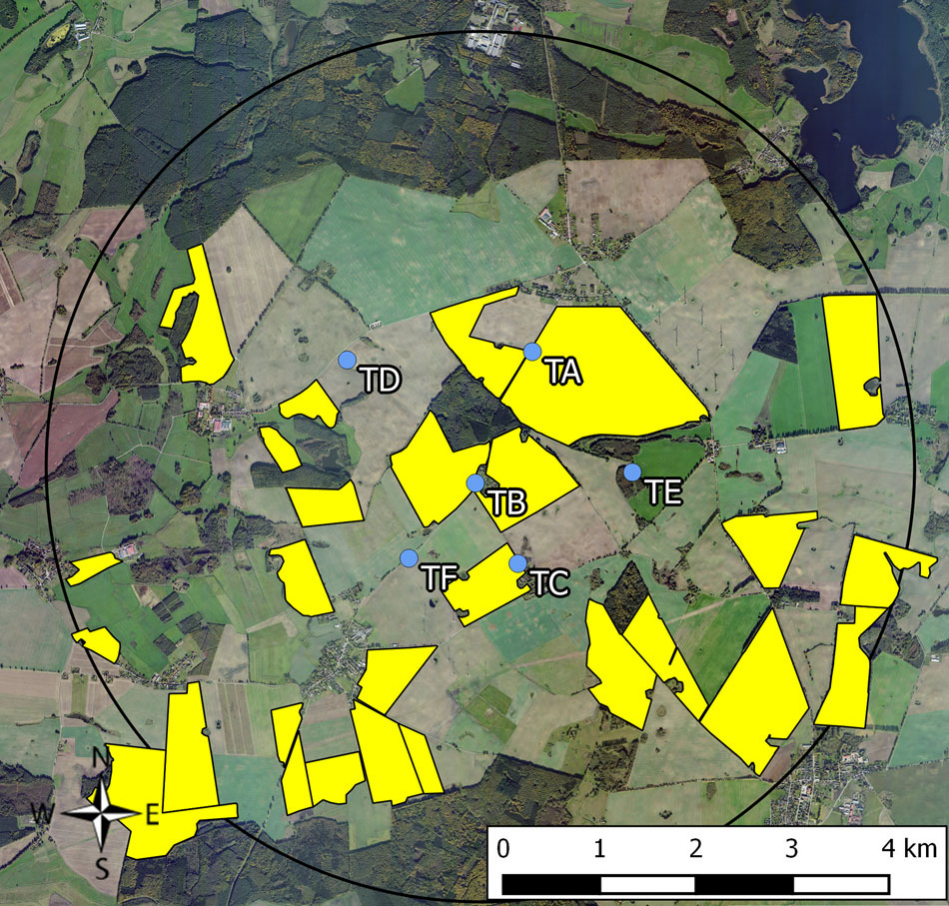
Test site (fields with treated seeds) used for the monitoring of effects of flowering OSR grown from clothianidin-dressed seeds. Study locations are marked in *blue*, *yellow polygons* indicate the study fields

**Fig. 3:**
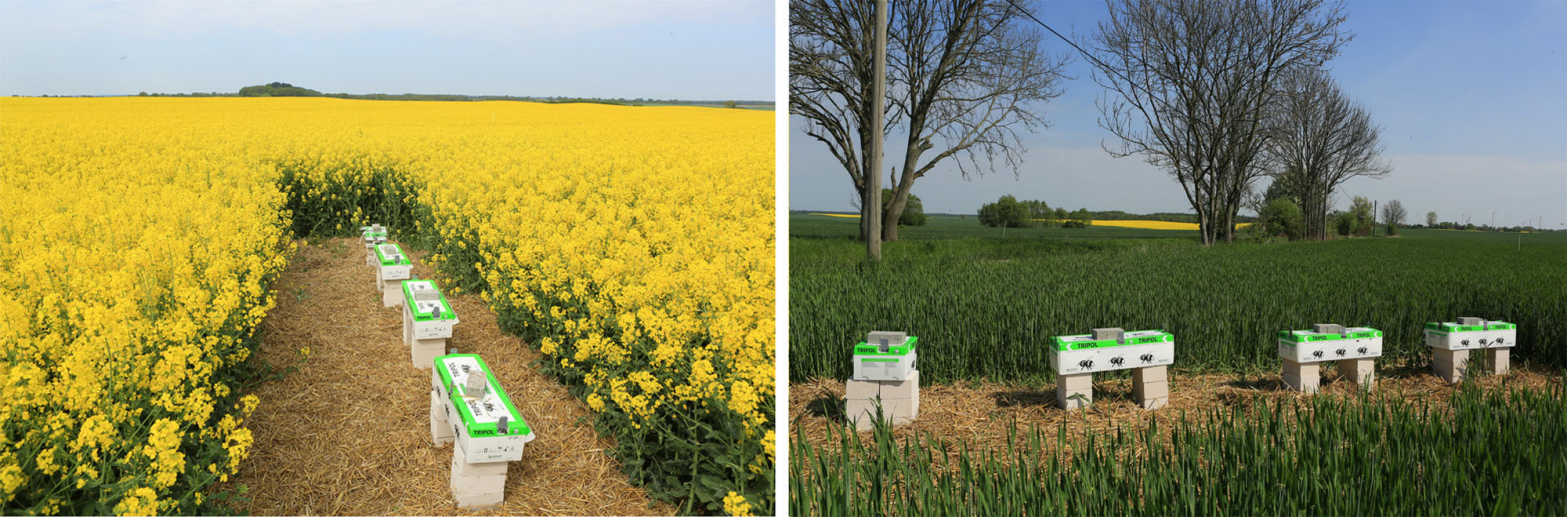
Photographs illustrating the placement of the bumble bee hives that were monitored in order to test the effects of flowering OSR treated with clothianidin seed dressing. Left: Hives placed at the edge of OSR fields (Note that a small clearing was cut in the fields to place the hives, the view in this picture is from the field edge onto the field). Right: Hives placed distant (400 m) to the OSR fields

The study was conducted in 2014 in Mecklenburg-West Pomerania, Germany. In this region, OSR is usually cultivated at 25–33 % of the arable land. Two circular study sites of approx. 65 km^2^ were selected: a reference site without clothianidin OSR seed dressing (Fig. 1) and a test site (Fig. 2) with a commercial dressing containing clothianidin (Elado^®^: 10 g clothianidin, 2 g beta-cyfluthrin/kg seed). Except for the clothianidin dressing of OSR seeds at the test site, no further neonicotinoids were used from autumn 2013 until summer 2014 at all study fields. Before drilling in autumn 2013, soil samples were collected from all study fields for the analysis of clothianidin residues and soil characterization (for details of farming, and PPP applications and soil analysis in the study area, see: Heimbach et al. 2016). The six study locations were situated in the core area of each of the two study sites, which should ensure that all OSR fields within the foraging distance of the bumble bees were either with or without clothianidin seed dressing. In order to test if potential effects interacted with the distance to the treated fields, at three of the six locations per study area the bumble bee hives were established directly at the edge of OSR fields, while the hives at the other three locations were situated ca. 400 m from the nearest OSR field (Fig. 3).

Detailed descriptions of the sites, seed treatment and planting are given in Heimbach et al. (2016). At each study location three multi-hives, called tripols (and one additional single hive for pollen collection, resulting in a total number of 10 hives per location) were placed facing south at the beginning of OSR full flowering (63–65 on the extended BBCH-scale coding of phenologically similar growth stages of all monocotyledonous and dicotyledonous plant species).

Tripols were assigned randomly to treatment and location. The entrance hole of each colony was fitted with a small plastic shutter, a so-called queen locker, which prevented young queens from leaving the hive.

The hives used for this experiment differed substantially from hives which are usually commercially available. In order to achieve a high comparability, every hive consisted of a mother queen from the same hibernation batch and 40 to 50 workers (a number which we considered as sufficient for the colonies to maintain temperature during cold weather) of roughly the same age. Workers that hatched on the same day were selected by hand to form very homogenous colonies. As workers may die during transport and handling, hives were checked before the start of the experiment and only the most homogenous ones with a healthy queen were used in the field. Each hive was provided with a sealable bag of sugar solution which was opened only when hives were closed during transport to the study locations. The three tripols per site (=9 colonies) were used for the regular assessments and the additional single hives for collection of pollen.

The exposure phase lasted 22 days, starting on 24 April 2014 with the placement of the tripols (**D**ay **A**fter **P**lacement 0) and ending with the end of OSR bloom and the subsequent relocation of the hives from the fields to a nature park in Belgium (Park Lieteberg, which belongs to the National Park Hoge Kempen, covering more than 5700 ha and consisting mainly of forests, lakes and heath) in May 2014 (DAP 24–26).

Relocation of the colonies started after most colonies had reached the turning point, which marks the cessation of the production of new workers and the first appearance of young queens and drones. The post-exposure phase lasted 21 days. The last assessments for the post-exposure phase were finished by June 2014 (DAP 43).

### Assessments during the exposure phase

During the exposure phase, regular assessments were performed twice a week, starting on 26 April 2014. Hives were removed from the tripols, and searched for the original mother queen before the hive was weighed. Closed hives were weighed using a platform balance with an accuracy of ± 5 g. The number of workers was estimated according to a categorization system (Table 1). Assessments were performed during daytime; hives were closed for the estimation. It was assumed that the number of foraging bumble bees during the assessments was more or less the same, so no correction for their numbers was employed. Special attention was also given to the behaviour of the bumble bees. Abnormalities in flight activity as well as guarding and cooling behaviour were recorded. All assessments were conducted by the first author, a person with many years of experience in bumble bee research.

**Table 1:** Categories used to estimate the number of worker bumble bees as used for the monitoring of effects of flowering OSR grown from clothianidin dressed seeds on bumble bees

Category	Scale (Number of individuals)	Category means
1	0–5	2.5
2	6–10	8
3	11–16	13.5
4	16–20	18
5	21–30	25.5
6	31–40	35.5
7	41–50	45.5
8	51–75	63
9	76–100	88
10	101–125	113
11	126–150	138
12	151–200	175.5
13	201–300	250.5
14	300+	350.5 (if using upper bound 400)

Pollen for identification was sampled twice at every study location during OSR flowering from single hives. The first sampling event took place between 28 and 30 April 2014 (DAP 4–6) and the second between 9 and 12 May 2014 (DAP 15–16). The BBCH of the OSR was 65 at the first and 67 at the second sampling event. To obtain the samples, returning bumble bee workers with pollen loads of the hive that was solely designated to pollen collection were caught with a vacuum collector containing dry ice. Approximately 20 returning foragers with pollen loads were collected on the first and approximately 40 on the second sampling event. Sampled bumble bees were put in a cooling box on dry ice; pollen loads were picked from the legs of the bees and stored at −18 °C until microscopical evaluation.

Pollen samples were unfrozen in the laboratory, suspended in distilled water and heated to dryness on a microscope slide. After glycerine was added to the samples the pollen composition was analysed by the use of microscopes (magnification factor 400× to 1000×). Pollen grains were determined as far as taxonomically practicable and an estimate of the quantity of each pollen species was conducted by dividing the field of vision into a matrix and counting sub-samples.

Pollen for residue analysis was sampled from returning foragers once (DAP 20) at each location. A detailed description of pollen residue analysis is provided in Rolke et al. (2016b).

Temperature, humidity, rainfall and wind conditions were recorded at each location. Detailed methodologies for climate recordings as well as results are given in Heimbach et al. (2016).

### Assessments after the exposure phase

Assessments for the presence of a healthy queen, the hive weight and the number of workers were conducted once a week during the post-exposure phase similar to the methodology during the exposure phase. In June, at the end of the life time of the colonies (i.e., after the turning point of the colonies was reached and no more workers were produced), the hives were frozen and dissected. All hives had been equipped with queen lockers that prevented queens from leaving the colonies, so that the total number of queens produced by a hive could be estimated by the end of the experiment. Queens were sorted and queen brood cells were estimated.

### Statistical evaluation

Generalized Linear Mixed Models (GLMMs) and Linear Mixed Models (LMMs) provide a flexible tool for analysing non-normal and normal data when independence of measurements is violated by spatial or temporal grouping of measurements. GLMMs and Generalized Additive Mixed Models were used to study the fixed effects of treatment and weather conditions, while the study location and the individual hive were incorporated as random effects. In addition, Beta Regression Models were used.

Poisson GLMMs with observational level random effects were fitted to the count data of reproductive endpoints. LMMs with a quadratic term of DAP were fitted to the hive weight data from all assessments and from assessments in the exposure phase. The model used for this statistical analysis excluded temperature sum and humidity sum as predictor variables because of their high multicollinearity with the DAP. For the development of the number of workers, the categories were transformed into mean numbers and fitted using standard mixed models. For the pollen composition data, beta regression and logistic regression models were fitted to the relative amount of OSR pollen. Because beta regression requires data to be strictly greater than 0 and smaller than 1, values of 100 % were corrected to 99.9999 % before fitting the model.

Statistical evaluation was conducted with the statistical software package “R” (version 3.0.1, R Development Core Team, Vienna, Austria, 2013). GLMMs were fitted to the data by using the packages “lme4” (Bates et al. 2015) and “nlme” (Pinheiro and Bates 2015). For multiple comparisons of parameters the package “multcomp” (Hothorn et al. 2008) was applied.

The minimum detectable difference (MDD) concept has been developed as an indicator of the power of a test *a posteriori* for aquatic mesocosm/microcosm studies (Brock et al. 2014). However, its calculation depends on the statistical analyses (or tests) applied to analyse the data. The calculation of the MDD for the monitoring study extended the MDD concept to suit the mixed model analysis. Augmented prediction confidence intervals were used as the basis for the derivation of the MDD and MDD%.

## Results

### Pollen composition

**Fig. 4:**
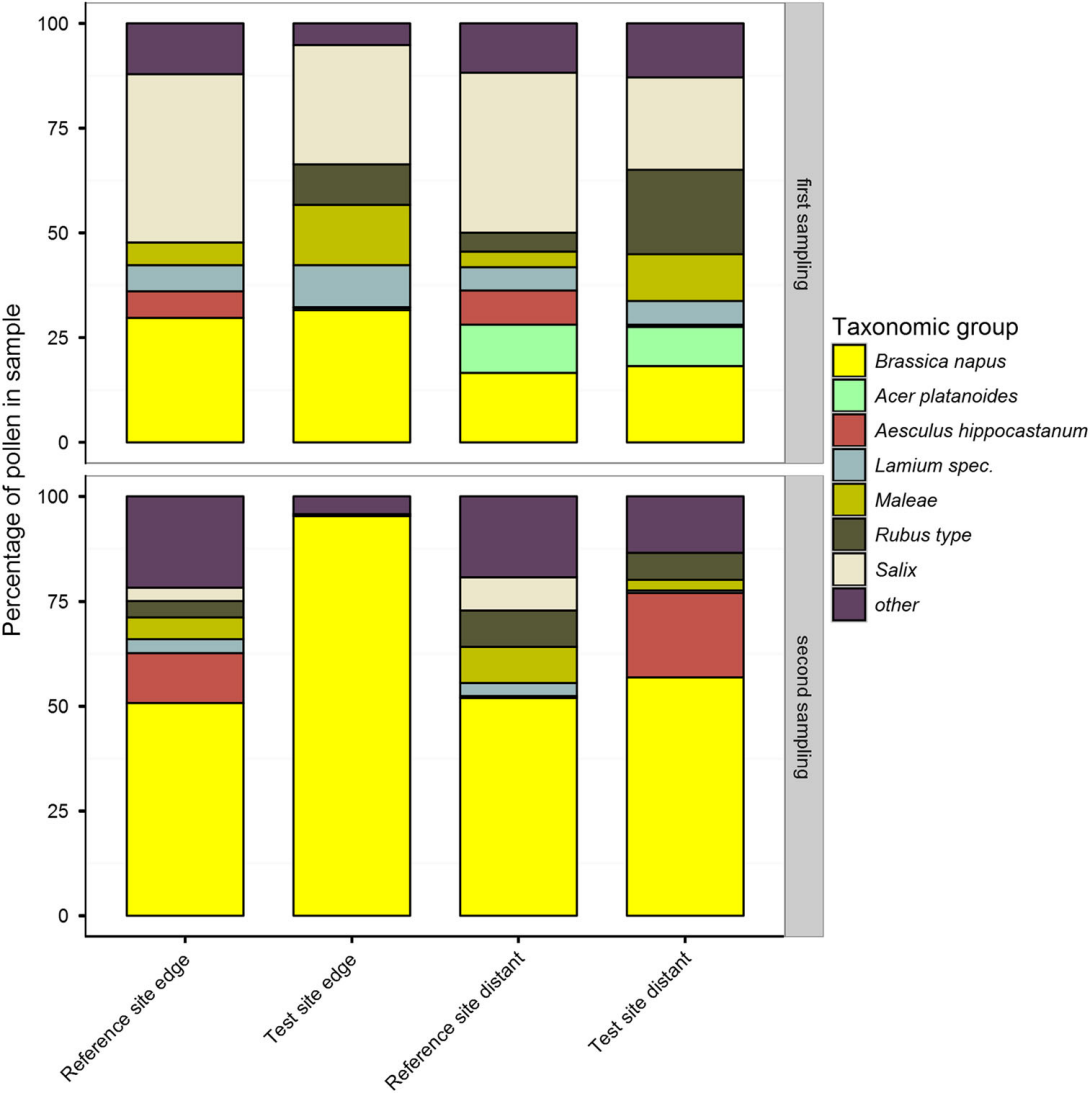
Composition of pollen samples. Bumble bee hives were either placed in a landscape with OSR fields treated with clothianidin seed dressing (test site) or untreated fields (reference site). Hives were either placed at the edge of the fields (edge) or ca. 400 m distant from the fields (distant). At each site one hive was designated to the collection of returning foragers carrying pollen. Two sampling events were conducted. The mean percentage of the different pollen species for each experimental group is shown. Pollen species representing less than 5 % of the total amount at the reference site were grouped as ‘other’

The mean amount of OSR pollen varied between 16 % (reference site distant) and 32 % (test site edge) in the first sampling (DAP 6, Fig. 4). Other important pollen sources at the time of sampling were trees of the genus *Salix* and Rosaceae (*Rubus* and *Maleae*), which are very attractive to bumble bees. Pollen samples taken during the second sampling (DAP 16) showed an OSR pollen content of 51 % (reference site edge) to 95 % (test site edge) indicating that OSR was the most important pollen source at this time*. Aesculus hippocastanum* also provided a major pollen source, but only at two of the four study locations. The proportion of OSR pollen collected by bumble bees differed between reference and test site, as well as between the first and second sampling reflecting the natural variability of the study area. Bumble bees collected significantly more OSR pollen at the test site (*p* = 0.020) than in the reference site, when combined values for both sampling events were considered. This is possibly due to the larger OSR area at the test site as compared to the reference site. When comparing the sampling events, significantly more OSR pollen was collected at the second sampling (*p* < 0.001) (Table 2) reflecting the increasing importance of OSR as a resource for bumble bees over time.

**Table 2:** Statistical evaluation of OSR pollen concentration in pollen pellets collected by bumble bee colonies placed in a landscape with flowering OSR grown from clothianidin treated seeds or in a landscape with OSR fields without clothianidin seed treatment

	Amount of OSR
Intercept	−1.53 ± 0.40 (<0.001)
Treatment	1.00 ± 0.43 (0.020)
2nd sampling event	1.97 ± 0.46 (<0.001)

Summary of the result from the beta regression model ‘Relative amount of *Brassica napus* (OSR) pollen’. Positive values indicate positive interaction, negative values indicate negative interaction, *p*-values in brackets. The intercept is the estimated mean value of the dependent variable, when all continuous variables are held at 0 and all categorical variables are held at their baseline levels. ± Standard deviation

### Residues

No clothianidin residues in pollen were quantified at the reference site (<LOD), whereas measurable residues were found in three locations at the test site: 1.0 at two locations and 1.3 µg/kg at one location. At the other three locations of the test site, clothianidin concentrations were below the LOQ (<1.0 µg/kg). It is not possible to relate the amount of OSR pollen to the residues in the pollen since samples were not collected at the same day. The concentrations of the two metabolites thiazolylmethylurea and thiazolylnitroguanidine (TZNG) in pollen pellets were below the LOD with the exception of one study location at the test site where the value for TZNG was below the LOQ. Further details are presented in Rolke et al. (2016b).

### Hive weights

**Fig. 5:**
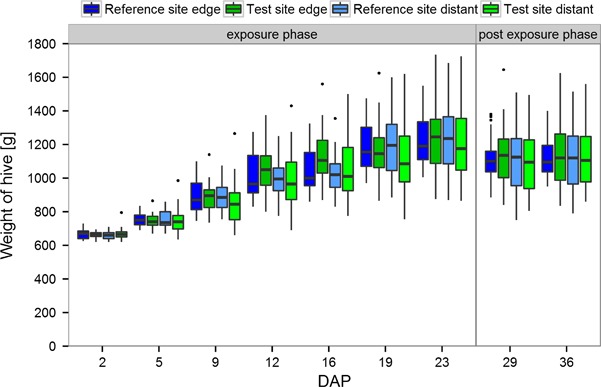
Development of bumble bee hive weights. DAP: Days after Placement. After the exposure period colonies were relocated to a nature reserve area for further monitoring. The upper and lower hinges of the boxplot correspond to the first and third quartiles (the 25th and 75th percentiles). Whiskers extend from the highest value to the lowest value within 1.5 times the interquartile range. Data beyond the end of the whiskers are plotted as points

The development of hive weight at all sites showed a continuous increase during the exposure phase (DAP 2–19). Two colonies at a distant location in the reference site were found without a queen on DAP 16. As these colonies were identified by exploratory data analysis (boxplots statistics) as outliers, they were excluded from all further evaluations. While these colonies developed poorly, the hive weight gain of all other colonies indicated that resources were adequate for proper colony growth. After the colonies reached their turning point and their subsequent removal from the study sites, no further gain of hive weight was observed (Fig. 5).

Weather conditions (wind speed and rainfall) had a statistically significant effect on the hive weight. No statistically significant treatment effect was detected (*p* = 0.944, Table 3) although the experimental design of the study would have been able to identify even small treatment effects if present as indicated by the MDD% (ranging from 5.7 to 10.9) and the relative deviations of the predicted test site mean hive weights from the reference site mean hive weights (Table 4).

**Table 3:** Statistical significances of the influence of different factors on colony development for bumble bee colonies placed in a landscape with flowering OSR grown from clothianidin treated seeds or in a landscape with OSR fields without clothianidin seed treatment

	Hive weight^a^	No. of workers
Intercept	571.81 ± 6.47 (<0.001)	56.35 ± 5.97 (<0.001)
DAP	37.12 ± 2.13 (<0.001)	6.19 ± 1.44 (<0.001)
Treatment	0.56 ± 7.77 (0.944)	−0.87 ± 6.31 (0.893)
DAP^2^	−0.76 ± 0.03 (<0.001)	−0.15 ± 0.01 (<0.001)
Distance to OSR 400 m	−3.39 ± 6.85 (0.633)	−8.03 ± 5.87 (0.204)
Temperature (sum)	–	0.091 ± 0.09 (<0.001)
Humidity (sum)	–	0.14 ± 0.02 (<0.001)
Wind speed (sum)	4.68 ± 1.02 (<0.001)	0.48 ± 0.24 (0.043)
Rainfall (sum)	0.92 ± 0.35 (0.009)	0.01 ± 0.14 (0.944)
DAP: Treatment	−2.84 ± 1.95 (0.145)	0.10 ± 0.15 (0.517)
Treatment:DAP^2^	0.07 ± 0.04 (0.070)	–

Summary of Poisson GLMM results; Hives CE-2-2 and CE-3-2 as outliers were excluded from calculations. The intercept is the estimated mean value of the dependent variable, when all continuous variables are held at 0 and all categorical variables are held at their baseline levels. DAP^2^ (i.e., the quadratic term of DAP) is included in the model because exploratory data analysis indicated a quadratic relationship (a parabolic curve) between DAP and hive weight or the number of workers, respectively. Positive values indicate positive interaction, negative values indicate negative interaction, *p*-values in brackets. ± Standard deviation

^a^ Temperature sum and humidity sum are all important factors (*p* < 0.001). They are also highly correlated with each other as shown in the correlation and variance inflation factor analysis. The coefficient estimates of these factors, however, should be interpreted with caution, as it is not possible to accurately describe the influence of single factors on the model when correlation between them occurs. Rainfall sum is an important factor for hive weight, but it does not significantly influence the number of workers

**Table 4:** Summary table of MDDs (Minimum Detectable Differences), Relative MDDs (MDD%), and relative differences (%) for various measures of bumble bee development and reproduction for bumble bee colonies placed in a landscape with flowering OSR grown from clothianidin treated seeds or in a landscape with OSR fields without clothianidin seed treatment

	MDD	MDD%	Relative difference (%)
Hive weight^a^	44.6 – 121.3^b^	5.7 – 10.9^b^	−2.4 – −0.7^b^
No. of workers	65.8 – 66.2^b^	48.8 – 186^b^	−1.2 – 7.3^b^
No. of young queens	35.6 – 51.3^c^	45.1 – 45.3^c^	−6.1^c^
No. of queen brood cells	20.5 – 21.9^c^	43.7 – 43.8^c^	+114^c^
Sum of young queens and queen brood cells	49.9 – 58.5^c^	36.0^c^	+46.5^c^

Positive values (for the relative difference) indicate that the response was enhanced for the test site, negative values the opposite

^a^ Model excluding temperature sum and humidity sum as predictors

^b^ Maximum and minimum for the comparison of reference site (edge, distant) and test site (edge, distant) for each DAP

^c^ Maximum and minimum for the comparison of reference site (edge, distant) and test site (edge, distant)

### Number of worker bumble bees

**Fig. 6:**
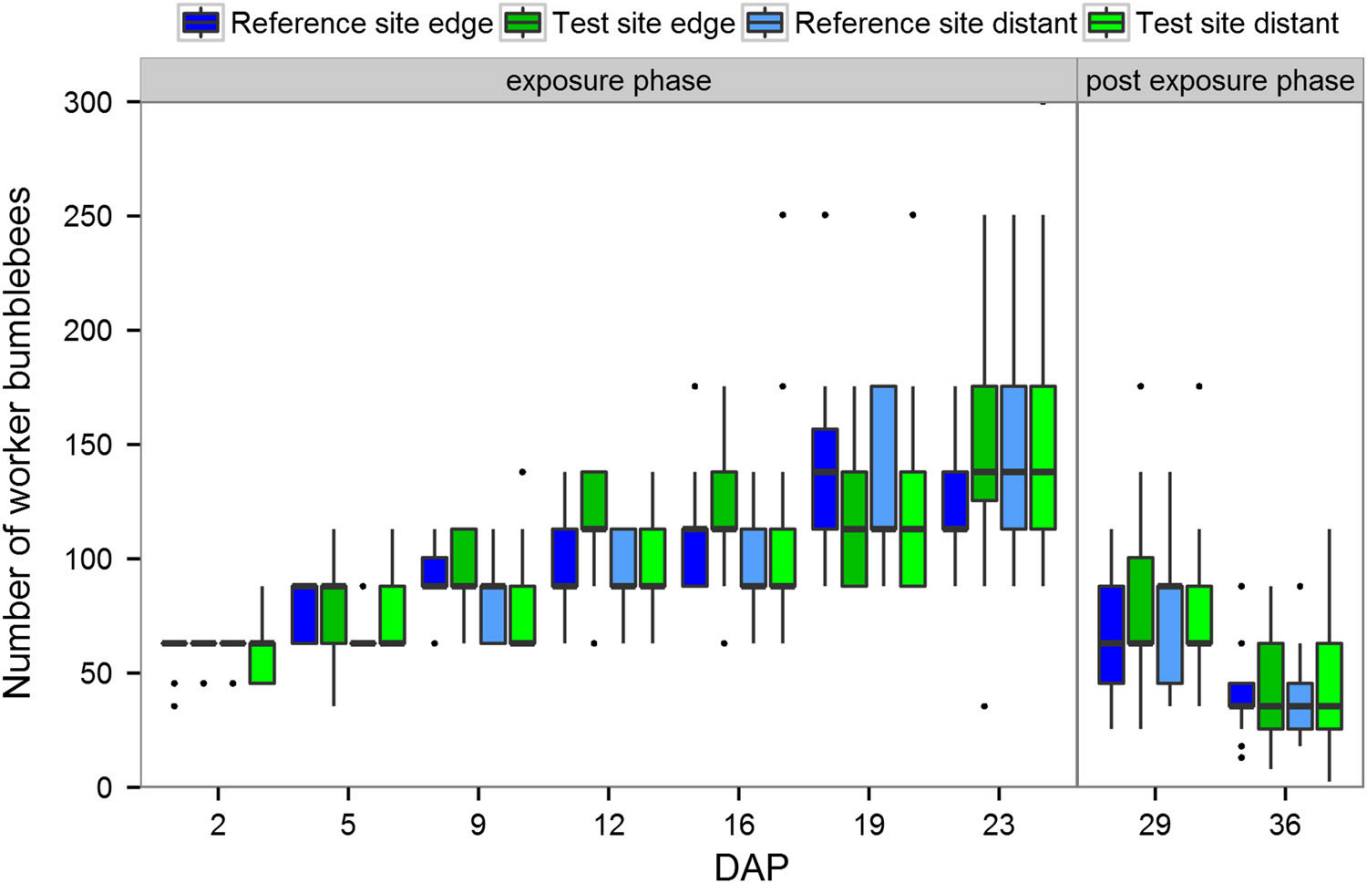
Number of bumble bee workers as estimated by category. Bumble bee hives were either placed in a landscape with OSR fields treated with clothianidin seed dressing (test site) or untreated fields (reference site). Hives were either placed at the edge of the fields (edge) or ca. 400 m distant from the fields (distant). DAP: Days after Placement. After the exposure period colonies were relocated to a nature reserve area for further monitoring. The upper and lower hinges of the boxplot correspond to the first and third quartiles (the 25th and 75th percentiles). Whiskers extend from the highest value to the lowest value within 1.5 times the interquartile range. Data beyond the end of the whiskers are plotted as points

The number of worker bumble bees at the first assessment was very similar for all sites and locations. As the colonies had been equalized for the numbers of worker bumble bees, this showed that this equalization was successful and all colonies had started the experiment at the same level. Subsequently, the number of worker bumble bees steadily increased during the exposure phase (Fig. 6) reaching its peak on DAP 23. Development of the colonies was homogenous for all sites. Assessments after DAP 23 (during the post-exposure phase) showed a decline in the numbers of workers, confirming that colonies had reached the turning point, after which colonies ceased to produce worker bumble bees.

Statistical analysis showed an influence on the number of worker bumble bees from weather conditions (temperature, humidity, wind speed), but no significant effect of the treatment was found (Table 3). The MDD % ranged from 49 to 186 % (Table 4). Due to the categorization system which could only characterize the approximate number of workers, small differences could not be identified. Therefore, the data collected cannot be considered as precise numerical values and the conducted hypothesis testing for the treatment effect on the development of worker numbers was not able to detect small differences. However, relative differences between hives from the reference and the test site ranged from −1.2 % to 7.3 % only. Therefore, it can be concluded that even though the statistical analysis was not able to detect a small difference due to the experimental design, no adverse treatment effects were observed from the data.

### Reproduction

**Fig. 7:**
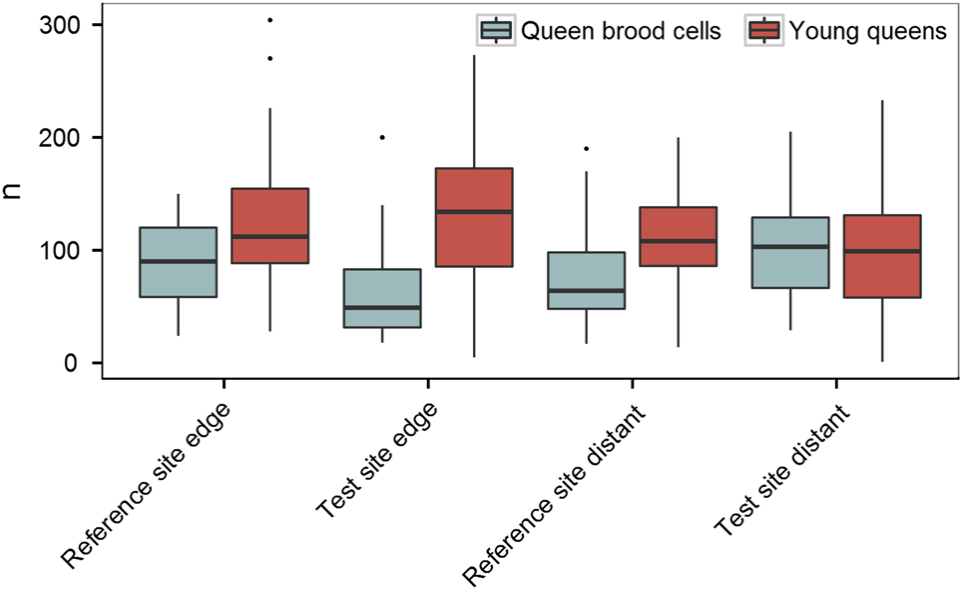
Numbers of young queens and queen cells at the end of the experiment. Bumble bee colonies were frozen, and the number of young queens and queen cells were determined. Bumble bee hives were either placed in a landscape with OSR fields treated with clothianidin seed dressing (test site) or untreated fields (reference site). Hives were either placed at the edge of the fields (edge) or ca. 400 m distant from the fields (distant). During the study, all hives were fitted with a queen lock to prevent young queens from escaping, so that a reliable determination of their number was possible at the end of the experiment. The upper and lower hinges of the boxplot correspond to the first and third quartiles (the 25th and 75th percentiles). Whiskers extend from the highest value to the lowest value within 1.5 times the interquartile range. Data beyond the end of the whiskers are plotted as points

The first colonies reached the turning point between DAP 19 and DAP 23. At DAP 23, young queens were found at all study locations. On DAP 23 the majority of the 54 colonies per treatment had reached the turning point. In detail, 37 (19 edge and 18 distant) colonies at the reference site and 41 (23 edge and 18 distant) at the test site were found to have started with the production of new queens. Figure 7 presents the numbers of new queens as well as the counted queen cells for the different sites and distances. The mean number of young queens at the end of the experiment ranged from 95 (test site distant) to 128 (reference site edge). These comparatively high numbers for commercially bred bumble bee colonies are indicative for the good status of the colonies. Taking the sum of young queens and queen brood cells can be considered as a measure for reproductive success, which ranged from 182 (reference site distant) to 216 (reference site edge).

Hives at the test site produced significantly more queen brood cells (*p* = 0.035). Significantly (*p* = 0.021) fewer young queens were found at study locations 400 m distant from the OSR fields, as compared to study locations at the edge of OSR fields (Table 5). However, the sum of queen brood cells and young queens as an overall indicator of reproductive success of bumble bee colonies was neither significantly influenced by the treatment nor by the distance of the hives to the OSR fields.

**Table 5:** Statistical significances of the influence of different factors on the reproduction endpoint for bumble bee colonies placed in a landscape with flowering OSR grown from clothianidin treated seeds or in a landscape with OSR fields without clothianidin seed treatment

	Number of young queens	Number of queen brood cells	Sum of young queens and queen brood cells
Intercept	5.25 ± 1.07 (< 0.001)	7.15 ± 1.03 (< 0.001)	6.73 ± 0.80 (< 0.001)
Treatment	−0.06 ± 0.38 (0.868)	0.76 ± 0.36 (0.035)	0.38 ± 0.28 (0.172)
Distance to OSR (400 m)	−0.37 ± 0.16 (0.021)	0.06 ± 0.15 (0.679)	−0.16 ± 0.12 (0.179)
Temperature (sum)	0.16 ± 0.19 (0.399)	0.22 ± 0.19 (0.234)	0.20 ± 0.15 (0.171)
Humidity (sum)	0.13 ± 0.11 (0.248)	−0.11 ± 0.11 (0.279)	0.03 ± 0.08 (0.712)
Wind speed (sum)	0.19 ± 0.08 (0.018)	0.09 ± 0.08 (0.250)	0.12 ± 0.06 (0.038)
Precipitation (sum)	−0.01 ± 0.03 (0.676)	−0.07 ± 0.02 (0.005)	−0.03 ± 0.02 (0.074)

Summary of Poisson GLMM Results; Hives CE-2-2 and CE-3-2 as outliers excluded from calculation. The intercept is the estimated mean value of the dependent variable, when all continuous variables are held at 0 and all categorical variables are held at their baseline levels. Positive values indicate positive interaction, negative values indicate negative interaction, *p*-values in brackets. ± Standard deviation

Statistical analysis showed that weather conditions (e.g., wind speed or humidity) influenced reproductive parameters (Table 5). The number of new queens was positively influenced by wind speed, the same effect was observed for the sum of young queens and brood cells. A negative influence on the number of queen brood cells was found for precipitation. However, as all the weather variables are probably correlated, these findings have to be treated with caution.

The MDD% for the number of queen cells ranged from 43.7 to 43.8, the MDD% for the number of young queens ranged from 45.1 to 45.3 and was 36.0 for the sum of young queens and queen brood cells.

### Behaviour

No abnormalities in behaviour, such as apathy or a lack of flight activity, were observed at any time in any hive. Furthermore, highly specialised behaviours like cooling of hives or guarding by young workers were regularly observed during the study at hives at both study sites.

## Discussion

In this monitoring study at the landscape level, OSR pollen was an important resource for all bumble bee colonies at both reference and test site. More OSR pollen was found during the second sampling event and more OSR pollen was found at the test site in comparison to the reference site. Despite the pollen sample collected at the test site edge during the second sampling event almost exclusively consisted of OSR pollen, this does not necessarily implicate that foraging bumble bees preferred treated OSR. First of all, acreage of OSR was twice as large at the test site in comparison to the reference site, which could have led to a simple stochastical effect. Furthermore, while no other flowering crops attractive to bumble bees were present, it was not feasible to control for other resources, such as hedgerows, flowering fruit trees in gardens etc. It is possible that foraging behaviour was influenced by the availability of alternative resources, which were not evenly distributed at both sites. Bumble bees are known to show a complex foraging behaviour influenced by the distribution and availability of the floral resources in the landscape (Goulson 1999). It has been shown previously that foraging bumble bees, even in the presence of rewarding resources close to the nest, will also use other resources which require longer flight distances (Osborne et al. 1999; 2008). Goulson et al. (2002) could show that the diversity of pollen loads was lower in agricultural landscapes in comparison to other habitats. When the abundance of other flowering plants is low, bumble bees will mainly use the most abundant resource, which in this study was OSR.

The main aim of pollen sampling was to show that foraging bumble bees did not avoid treated OSR, which is supported by our data. Beyond this, we did not closely monitor the development of bumble bee foraging behaviour over time (not least because of a lack of suitable methods to do so).

Residue analysis showed that test site colonies were indeed exposed to clothianidin (1.0–1.3 µg/kg was measured in three study locations while in the other three locations residues were below the limit of quantification), whereas no evidence for exposure to clothianidin was found for reference site colonies (see: Rolke et al. 2016b, for details).

In conclusion our data reliably show that bumble bees were foraging on the treated crop and were exposed to clothianidin. Further investigations of the foraging behaviour are beyond the scope of this study.

Our results did not show any adverse effect of clothianidin-treated OSR on bumble bee colony development as measured in terms of hive weight. Furthermore no effect of clothianidin-treated OSR was detected considering the numbers of worker bumble bees. While this assessment of colony development by an experienced expert provides a non-invasive, non-destructive method to assess the number of individuals in a bumble bee colony over time, it is less well suited for detecting small differences in bee numbers and, therefore, the resulting endpoint is biologically less meaningful. The (statistically) more powerful results describing the development of bumble bee hives are hive weight and numbers of young queens and queen brood cells.

No effect was observed on numbers of young queens and queen cells, which is the biologically most meaningful endpoint. Effects on overwintering and breeding success were not directly measured as this would have required mating and hibernation of every new queen under controlled environmental conditions.

While queen weight has been recently proposed as endpoint determining colony fitness (Cabrera et al. 2016), we did not measure it in this study. Beside the fact that it would have been logistically challenging to measure the weight of all new queens in a large field study like ours there is a profound reason why only the number of new queens was measured. In order to account for all queens that hatched in a colony, queen lockers or excluders were attached to the entrance of each hive that prevented any queen from exiting. As new queens do not hatch simultaneously this means that some of the new queens would have stayed in the hives for a certain period without much pollen or nectar. This would have created a bias in the queen weight as individuals that hatched early would have lost already some of their hibernation fat.

Some earlier studies reported negative effects of neonicotinoids on bumble bee colony development and reproduction (Whitehorn et al. 2012; Feltham et al. 2014). However, these studies often represent unrealistic worst-case scenarios. Whitehorn et al. (2012) and Feltham et al. (2014) exposed bumblebee colonies to comparatively high doses of imidacloprid and measured the impacts on colony reproduction and foraging behaviour, respectively. In these studies no alternative food sources besides the neonicotinoid containing food were offered for 14 days. Afterwards, effects were measured on free ranging colonies. We believe that unrealistic worst-case feeding studies under controlled conditions are not necessarily a good predictor for the effects of PPPs in complex field situations. The results of the present study indicate not only that exposure in the field is lower than often suggested (as shown by the residue levels found (maximum of 1.3 µg/kg in pollen collected by bumble bees) and the fact that alternative food sources were used, but also that no effect of the treatment on reproduction can be found under realistic exposure conditions.

In accordance with our findings, Thompson et al. (2013) found no relationship between the exposure to neonicotinoid treated OSR and bumble bee colony success in a field trial conducted in the United Kingdom. There are, however, several methodological problems with this work (e.g., differences in flowering phenology across the sites, which led to a delay in the placement of the bumble bee colonies at one of the sites making comparisons difficult). A recent statistical re-evaluation of the data has come to the conclusion that there were harmful effects (Goulson 2015). The uncertainty in the conclusions from such a study reflects the variability of field-based test systems and the current lack of established methodology.

A more comprehensive study has been carried out by Rundlöf et al. (2015), who monitored the effect of Elado^®^-dressed spring OSR on bumble bee colonies in a field trial in an agricultural landscape in Sweden. They reported a negative effect on bumble bee hive weights and reproduction (measured as the number of new queens). While this study is more comparable to our study, there are a number of relevant differences. Firstly, non-standardized hives were used in the study by Rundlöf et al. (2015), while we ensured that the hives used were uniform with regard to age and number of workers and queens that were from the same hibernation batch. Secondly, terminating the trial at the first sight of emerging new queens is not appropriate for such a study. Colonies that have a later turning point have been shown to produce even a significantly greater number of queens than colonies that reach the turning point early (Duchateau and Velthuis 1988). The hives should have been taken out of the fields and placed in a non-agricultural area until nearly all new queens were hatched. Thirdly, it is also unclear whether the use of other PPPs had had any influence on the outcome of the study by Rundlöf et al. (2015). An insecticide with the active substance indoxacarb has been sprayed in one treatment replicate the same day as bumble bee hives had been placed in the field. This substance is well known to be toxic to bumble bees, if they are exposed to wet spray residues on the crop up to 2 days after application (DuPont 2004; van der Steen and Dinter 2009).

In our study, however, indoxacarb was applied on seven reference fields at least 18 days before placement of bumble bee hives at the study fields. Thus, effects of the indoxacarb treatment on bumble bees can be excluded in our study since toxic effects are only to be expected up to 2 days after applications (van der Steen and Dinter 2009). All PPP applications during the exposure phase and shortly before placement of bumble bee hives have performed following label restrictions, excluding negative effects of PPP applications on bees. Lastly, different crops have been used in the study conducted by Rundlöf et al. (2015) and the present study. Spring OSR is sown in spring and flowers in the same year, while winter OSR flowers in the year after it has been sown. In addition, the latter has a prolonged flowering period in comparison to spring OSR. However, as discussed by Rolke et al. (2016b) the study conducted by Rundlöf et al. (2015) in Sweden has shown considerably higher residual concentrations of clothianidin in pollen and nectar collected by honey bees and bumble bees in Elado^®^ seed-dressed spring OSR (for further discussion, see: Rolke et al. 2016b).

In comparison to the current state of knowledge for bumble bees, more data is available for field studies with clothianidin treated OSR and honey bee colonies. Studies have been conducted in Canada, where long-term effects were assessed by exposing honey bee colonies to treated spring OSR and monitored until after overwintering (Cutler and Scott-Dupree 2007; Cutler et al. 2014). In the recent Swedish study, honey bee colonies were monitored during the exposure to treated spring OSR and shortly afterwards (Rundlöf et al. 2015). Furthermore, a large-scale monitoring study with winter OSR, which is described in detail in this issue (Rolke et al. 2016a), was conducted as part of this monitoring project. Interestingly, no effects on honey bee colonies have been found in any of these field studies. It has to be considered that commercial honey bees are domesticated animals, used to handling and regularly used in field experiments in the past. In contrast, the methodology for bumble bee studies is less well established. Different methods for the assessment of effects on the colony level might lead to different (even contrasting) results.

Repeating field and monitoring studies in subsequent years and/or other locations might be desirable in order to address remaining questions. However, a repetition of this monitoring study was not feasible because the logistics and demands of resources of such a big landscape study exceed common practices by far. Alternatively, certain aspects might even be addressed by specially designed lower tier studies.

## Conclusion

As monitoring studies at the landscape level are complex, difficult to conduct and cost-intensive, they are not regularly performed for the assessment of risk of PPPs to pollinating insect species. However, they may lead to assessments that cannot be obtained from lower tier studies, e.g., in relation to realistic exposure levels and the relevance of the measured endpoints. This is particularly the case for bumble bees as standardized methods for testing are not yet available.

In conclusion, we found no evidence that a single cause such as the use of neonicotinoids can be held responsible for a decline in populations of pollinators such as bumble bees when several factors such as availability of resources (particularly in relation to intensive agriculture), pathogens and other factors play a role in the survival of pollinating insect species. By employing appropriate methods under conditions that were realistically representing field conditions, but also worst case assumptions (e.g., no untreated OSR was within the foraging range at the test site) at the landscape level, the results obtained in the present study showed no adverse effects on bumble bee colonies from the exposure to winter OSR treated with Elado^®^.
